# Is a persistent central canal a risk factor for neurological injury in patients undergoing surgical correction of scoliosis?

**DOI:** 10.1186/s13013-017-0133-z

**Published:** 2017-09-14

**Authors:** Steven Kyriacou, Yuen Man, Karen Plumb, Matthew Shaw, Kia Rezajooi

**Affiliations:** 1 Spinal Deformity Unit, Royal National Orthopaedic Hospital, Stanmore, UK; 2 The Royal National Orthopaedic Hospital, Stanmore, UK

**Keywords:** Scoliosis, Persistent central canal, Syrinx

## Abstract

**Background:**

Scoliosis patients with associated syringomyelia are at an increased risk of neurological injury during surgical deformity correction. The syrinx is therefore often addressed surgically prior to scoliosis correction to minimize this risk. It remains unclear if the presence of a persistent central canal (PCC) within the spinal cord also poses a similar risk. The aim of this study is to determine whether there is any evidence to suggest that patients with a PCC are also at a higher risk of neurological injury during surgical scoliosis correction.

**Methods:**

Eleven patients with a PCC identified on pre-operative magnetic resonance imaging who had undergone correction of adolescent idiopathic scoliosis (AIS) over a 7-year study period at our institution were retrospectively identified. The incidence of abnormal intra-operative spinal cord monitoring (SCM) traces in this group was in turn compared against 44 randomly selected age- and sex-matched controls with no PCC who had also undergone surgical correction of AIS during the study period. Fisher’s exact test was applied to determine whether there was a significant difference in the incidence of abnormal intra-operative SCM traces between the two groups.

**Results:**

Statistical analysis demonstrated no significant difference in the incidence of abnormal intra-operative SCM signal traces between the PCC group and the control group.

**Conclusions:**

This study demonstrates no evidence to suggest a PCC increases the risk of neurological complications during scoliosis correction. We therefore suggest that surgical correction of scoliosis in patients with a PCC can be carried out safely with routine precautions.

## Background

One of the most devastating potential complications of scoliosis correction surgery is iatrogenic neurological injury [1, 2]. Numerous factors have been implicated as increasing the risk of such a complication including the presence of abnormalities within the spinal cord [3]. The incidence of spinal cord pathology in paediatric patients with scoliosis has previously been reported to be between 3 and 20%, with pre-operative magnetic resonance imaging (MRI) demonstrating various intra-spinal abnormalities including syringomyelia, Chiari malformation, diastematomyelia, tethered cord and spinal cord tumours [1, 4, 5]. The mechanism of neurological injury arising from surgical correction of scoliosis can be from an instrument or implant striking the spinal cord, from a vascular injury related to the implant causing stretching or compression of vessels or from vascular compromise not directly related to the implant such as ischaemia secondary to hypotension [6].

Previous studies have demonstrated patients with spinal cord pathology undergoing surgical correction of scoliosis are at an increased risk of sustaining intra-operative iatrogenic neurological injury [1, 7–9]. However, to the authors’ knowledge, there has not been a published study addressing the question as to whether the presence of a persistent central canal (PCC) also poses an increased risk of intra-operative neurological injury during surgical correction of scoliosis. The aim of this study is to therefore address this question.

## Methods

The null hypothesis to be tested was defined as patients with a PCC are at an equal risk of developing intra-operative neurological complications during surgical correction of scoliosis as patients without a PCC.

In order to test this hypothesis, all patients who had undergone surgical correction of adolescent idiopathic scoliosis (AIS) over a 7-year period between June 2004 and October 2011 at our institution who had a co-existing PCC confirmed with routine pre-operative whole spine MRI were retrospectively identified using an electronic database and were included in the study. MRI was performed at 1.5 Tesla with a phased array coil, and image sequences included T1-weighted spin echo (SE) and T2-weighted fast SE (FSE) sagittal and axial images. All MRIs were reported by a consultant musculoskeletal radiologist.

A PCC was defined as a filiform or slit-like, centrally located, intra-medullary cavity of a maximum diameter of 4 mm [10] not communicating with the fourth ventricle and extending over at least two vertebral levels [11] in the absence of any co-existing neuro-axis abnormality (NAA) on neuro-radiological imaging potentially responsible for cerebrospinal fluid (CSF) flow disturbances, with no prior history of spinal trauma, spinal infections or previous spinal/neurosurgical intervention. Additional inclusion criteria was the use of intra-operative spinal cord monitoring (SCM) during the surgical correction of the spinal deformity.

Eleven patients in total met these criteria and were included in the study. Forty-four sex- and age-matched control group patients who had also undergone surgical correction of AIS during the same study period with no underlying NAA evident on pre-operative MRI screening were randomly selected from a list of 1150 patients using Stata/IC version 12 software (StataCorp, College Station, TX, USA). Therefore, in total, 55 patients were included in the study.

The pre- and post-operative neurological status as determined by clinical examination findings up to the time of 3-month outpatient follow-up, the type of deformity correction and the intra-operative SCM traces were identified in the medical records of each patient included in the study. The intra-operative SCM traces for each patient were analysed by the Department of Neurophysiology, with somato-sensory evoked potentials (SSEPs) being used throughout the surgery to monitor for potential neurological compromise. Deviation from baseline SCM traces was classified as either ‘Green’ (no trace change), ‘Amber’ (an event causing an indirect effect with partial trace change) or ‘Red’ (an event causing a direct effect with partial to complete trace loss).

The SCM equipment used to monitor intra-operative SSEPs during the study period were Nihon Kohden Neuromaster (Tokyo, Japan) and Nicolet Biomedical (Viking Madison, WI, USA).

In order to assess whether there was any significant difference in the incidence of abnormal intra-operative SCM traces between the PCC group and the control group, a Fisher’s exact test was performed given the categorical nature of the data, again using Stata/IC version 12 software. In order to calculate a Fisher’s exact test, a 2 × 2 contingency table was created using the results of the intra-operative SCM traces for each of the 55 patients included in the study. For the purposes of entering this data into the contingency table, the patients in the PCC group were subdivided into those with normal (‘Green’) and abnormal (‘Amber’ or ‘Red’) intra-operative SCM traces. The patients in the control group were similarly subdivided into those with normal and abnormal intra-operative SCM traces.

In addition to comparing the incidence of abnormal intra-operative SCM traces between the PCC and control group, a comparison was also made of the incidence of post-operative neurological deficit apparent on clinical examination between the two groups to assess for any difference.

## Results

**Fig. 1:**
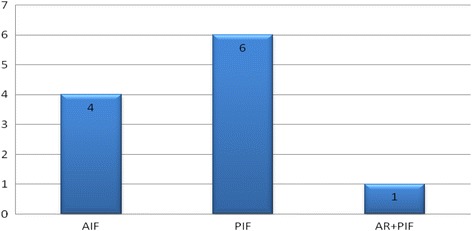
Type of scoliosis correction surgery performed on the 11 patients in PCC group. *AIF*–Anterior Instrumented Fusion, *PIF*–Posterior Instrumented Fusion, *AR*-Anterior Release

During the 7-year study period, 1161 AIS corrections were conducted out of which 11 patients met the criteria of having a PCC identified on pre-operative MRI. There was one male and ten females in the PCC group with an average age of 15.9 years (range 14–20). Only one patient in the PCC group had a pre-operative clinical neurological deficit in the form of mildly diminished sensation in the S1 distribution of the right foot with no associated motor weakness. No definite cause for this was demonstrated on pre-operative MRI and nerve conduction studies. Four patients in the PCC group underwent an anterior instrumented fusion (AIF), six patients underwent posterior instrumented fusion (PIF) and one patient underwent combined anterior release (AR) and PIF (Table 1 and Fig. 1).

**Table 1:**
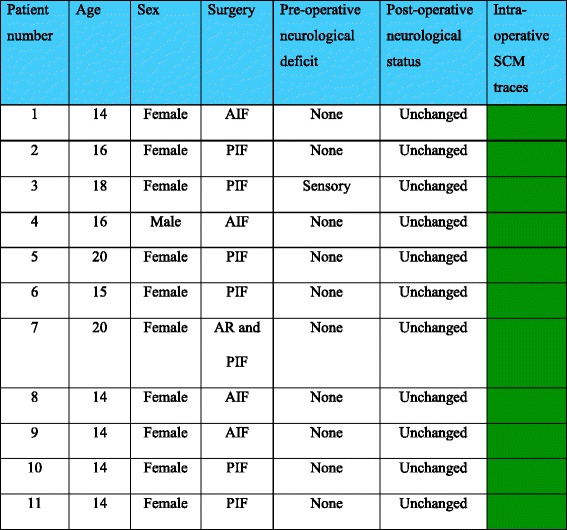
Demographic data and outcomes of patients in the PCC group

*AIF* anterior instrumented fusion, *PIF* posterior instrumented fusion, *AR* anterior release

**Fig. 2:**
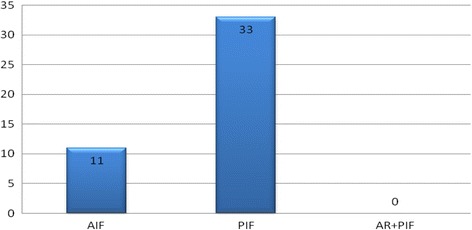
Type of scoliosis correction surgery performed on the 44 patients in the control group. *AIF*–Anterior Instrumented Fusion, *PIF*–Posterior Instrumented Fusion,* AR*-Anterior Release

There were 44 age- and sex-matched AIS controls. These included 4 males and 40 females with an average age of 15.9 years (range 14–20). Eleven patients in the control group underwent an AIF and 33 patients underwent a PIF (Table 2 and Fig. 2). No patient in the control group had a clinical neurological deficit pre-operatively.

**Table 2:**
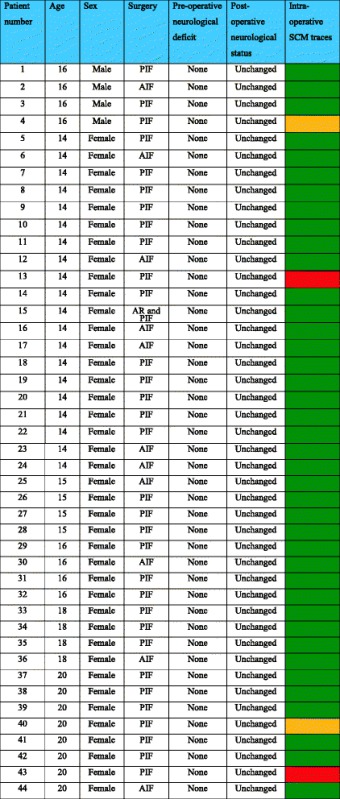
Demographic data and outcomes of patients in the control group

*AIF* anterior instrumented fusion, *PIF* posterior instrumented fusion, *AR* anterior release

**Fig. 3:**
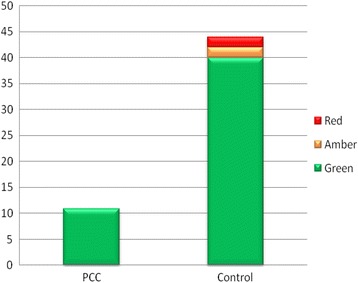
Number of patients with normal (*green*), borderline (*amber*) or abnormal (*red*) spinal cord monitoring traces observed in the persistent central canal group (*left*) versus the control group (*right*)

In both the PCC and control group, baseline SSEPs were obtained for all patients. In the PCC group, no patient had an intra-operative deviation from the baseline traces, compared to four (9.1%) patients in the control group. These four patients all had a PIF procedure. Of these four patients, two patients had ‘Amber’ warning signal changes intra-operatively and two patients had ‘Red’ warning signal changes intra-operatively (Fig. 3). In all four control group patients who developed abnormal intra-operative SCM traces, the traces returned to normal when appropriate intra-operative measures were taken to reverse the precipitating factor such as reducing the degree of distraction being applied to the spine. No patient in either the PCC or the control group had a new onset neurological deficit post-operatively evident on clinical examination.

The exact anatomical level of the PCC in each patient within the PCC group, the vertebral levels instrumented and the percentage deformity correction achieved were also recorded (Table 3). The PCC was found to be located either entirely or partially within the instrumented spinal levels in all patients within the PCC group. The average percentage deformity correction achieved was 70%.

**Table 3:** Level of PCC, curve pattern, level of instrumentation and percentage deformity correction achieved in PCC patients

Patient number	Level of PCC	Curve pattern	Levels instrumented	Pre-operative Cobb angle	Post-operative Cobb angle	% correction
1	T3-L1	Thoraco-Lumbar	T11-L3	52	12	77
2	T3-T8	Thoracic	T2-T12	55	20	64
3	C5-L1	Thoracic	T2-L1	59	22	62
4	T8-T12	Thoraco-Lumbar	T11-L3	54	19	65
5	C4-T3	Thoracic	T2-L2	61	15	75
6	C6-T4	Thoracic	T2-L1	62	19	69
7	T3-T9	Thoracic	T2-L2	69	25	64
8	T7-L1	Thoraco-Lumbar	T11-L3	54	12	78
9	T5-T12	Thoraco-Lumbar	T12-L3	52	15	72
10	T6-L1	Thoracic	T2-L3	57	15	74
11	T4-T8	Thoracic	T2-L3	60	21	65

*PCC* persistent central canal, *C* cervical, *T* thoracic, *L* lumbar

Fisher’s exact test did not demonstrate any statistically significant difference in the incidence of abnormal intra-operative SCM traces between patients in the PCC group and patients in the control group (*p* = 0.5728). The null hypothesis ‘patients with a PCC are at an equal risk of developing intra-operative neurological complications during surgical correction of scoliosis as patients without a PCC’ could therefore not be rejected.

## Discussion

**Fig. 4:**
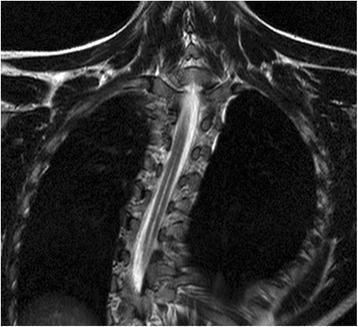
Image demonstrating typical appearance of a persistent central canal on T2-weighted coronal MRI of the thoracic spine

**Fig. 5:**
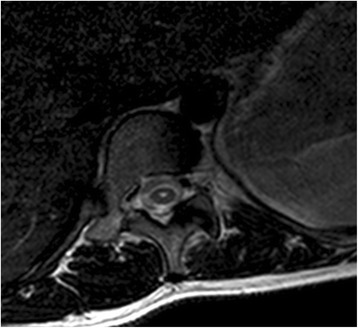
Image demonstrating typical appearance of a persistent central canal on T2-weighted axial MRI of a thoracic spine segment

The central canal is an ependymal-lined structure in the spinal cord that extends inferiorly from the fourth ventricle to the conus medullaris [12]. Anatomical studies suggest the central canal is only seen in foetal and newborn spinal cords and undergoes age-related stenosis such that it is obliterated in the vast majority of adults [13–16]. In a PCC, a degree of age related stenosis has occurred such that the central canal no longer extends all the way from the fourth ventricle to the conus medullaris. A partial remnant may persist, however, as shown in autopsy studies, and although reported to be seen in only 1.5% of MRI studies of the spinal cord, it can normally be regarded as an incidental finding [10, 14, 15]. The 4 mm maximum diameter used to form our definition of a PCC is based on the paper by Petit-Lacour et al. [10] which was the first study published describing the visibility of the central canal on MRI. The central canal can communicate with the fourth ventricle beyond infancy, but this is uncommon and is usually associated with hydrocephalus which excludes it from being a PCC which is essentially idiopathic. The typical appearance of a PCC on T2-weighted coronal and axial spinal MRI is demonstrated in Figs. 4 and 5, respectively.

The term ‘hydromyelia’ is often used to refer to an ependymal-lined, CSF-filled spinal cord cavity which most likely represents persistence into adulthood of a foetal configuration of the anatomy of the central canal of the spinal cord [17]. Hydromyelia can therefore be used interchangeably with PCC as they represent the same entity, although it could be argued that calling it a ‘persistent central canal’ is a more literal description.

In contrast to syringomyelia, the literature defining a PCC and determining its clinical significance remains limited. There is currently no widely accepted definition of a PCC in the literature, and debate continues on the criteria for distinguishing between a PCC and syringomyelia. Syringomyelia tends to be used to refer to a CSF-filled cavity within the spinal cord which is surrounded by a wall comprised of glial cells (which therefore implies it is related to a pathological process) and may present with abnormal neurological signs and symptoms. The term PCC is generally used to refer to an ependymal-lined, CSF-filled cavity within the spinal cord. They are usually asymptomatic and have no identifiable underlying cause. Much of the confusion arises from the fact that in practice, it is often not possible to distinguish between the two radiologically and therefore the umbrella term ‘syrinx’ is applied despite the fact this encompasses more than one entity.

Previous studies have recommended neurosurgical intervention should be performed prior to any orthopaedic procedure to reduce the higher risk of neurological injury associated with surgical correction of scoliosis in patients with neurological etiologies compared with patients with AIS [7–9, 18–24]. Whether or not a PCC should also be regarded as a risk factor for neurological injury during scoliosis correction is currently not clear and has to date not been addressed in the medical literature.

The general consensus is that a PCC may represent an anatomical variant with no identifiable underlying cause that is generally asymptomatic and most probably represents a different clinical entity from syringomyelia [11–13, 25]. Whether the presence of a PCC poses a risk of an individual ultimately developing syringomyelia in the future at present remains uncertain and open to discussion.

Of the 11 patients in the PCC group, all had ‘adolescent idiopathic scoliosis’, with the vast majority being female as would be expected in a group of patients with this condition. In order to minimise the risk of neurological injury during scoliosis correction, SCM is now routinely used during spinal deformity surgery and SSEPs represent the standard of care, their reliability in alerting the surgeon to a potential cord injury having been clearly established [26]. None of these patients had any intra-operative deviation from their baseline SCM traces or developed any postoperative neurological deficit as a complication of the surgical correction of their spinal deformity. There were no instances of any false negative SCM traces. When compared to their matched controls with regard to incidence of intra-operative deviation from baseline SSEPs, there was no statistically significant difference between the two groups (*p* = 0.57). Therefore, the null hypothesis could not be rejected, i.e. the presence of a PCC does not increase the risk of iatrogenic neurological injury during surgical correction of scoliosis.

The PCC was found to be located either entirely or partially within the instrumented spinal levels in all patients within the PCC group, and a satisfactory percentage curve correction was achieved in all 11 PCC patients. The absence of abnormal SCM traces in any of the 11 patients in the PCC group therefore cannot be attributed to either the PCC being anatomically remote to the level of instrumentation or be related to a minimal curve correction.

This study does have inherent weaknesses, the major one being the relatively small numbers involved. In order to increase the statistical power of the study, four times as many sex- and age-matched controls were included. However, the study spanned a 7-year period during which 1161 surgical scoliosis corrections were performed. This suggests that a PCC in a patient presenting with scoliosis is a rare finding, in the order of 0.95% in our study group. This is in a similar range to the 1.5% described by Petit-Lacour et al. [10], which at present remains the only published estimation of the prevalence of PCCs in the general population, based on a retrospective study of 794 whole spine MRI scans of patients who had initially been investigated for a variety of symptoms. Significantly increasing the numbers involved in our study would entail having to either lengthen the already considerable study period by a substantial amount of time or conduct a multicentre study, both of which have inherent difficulties.

Another weakness of this study is that it is retrospective and therefore has deficiencies inherent to all investigations of this nature. However, once again owing to the relative scarcity of PCCs, conducting a prospective study to address this research question is not practicable and is therefore very unlikely to ever be performed.

The significance of the results of this study relates to the fact that with the resolution of MRI scans progressively increasing and routine pre-operative MRI screening of all patients presenting with scoliosis becoming commonplace across a greater number of institutions, it is very likely that a growing number of patients with PCCs will be identified pre-operatively. At present, the pre-operative identification of a fluid collection within the spinal cord of a child with scoliosis but no other NAA often causes uncertainty for clinicians. The significance of such findings and the degree of additional risk of intra-operative neurological injury they may pose, if any, currently remains uncertain. Once identified, these patients are often pre-operatively referred for a neurosurgical opinion on the appropriate management of these entities. At present, this remains largely unknown due to the absence of any literature to guide clinicians in these circumstances. This is therefore an issue this study may help to address.

## Conclusion

Despite being based on relatively small numbers, our study does not provide any evidence to suggest that the presence of a PCC increases the risk of a neurological injury secondary to surgical correction of scoliosis. We therefore suggest that surgical correction of scoliosis in patients with a PCC can be carried out safely with routine precautions.

## Abbreviations

AIF: Anterior instrumented fusion
AIS: Adolescent idiopathic scoliosis
AR: Anterior release
CSF: Cerebrospinal fluid
MRI: Magnetic resonance imaging
NAA: Neuro axis abnormality
PCC: Persistent central canal
PIF: Posterior instrumented fusion
SCM: Spinal cord monitoring
SSEPs: Somato-sensory evoked potentials
